# Visualization of G-Quadruplexes, i-Motifs and Their Associates

**DOI:** 10.32607/actanaturae.11705

**Published:** 2022

**Authors:** E. V. Dubrovin, N. A. Barinov, D. V. Klinov

**Affiliations:** M.V. Lomonosov Moscow State University, Faculty of Physics, Moscow, 119991 Russia; Federal Research and Clinical Center of Physical-Chemical Medicine of Federal Medical Biological Agency, Moscow, 119435 Russia; Peoples’ Friendship University of Russia (RUDN University), Moscow, 117198 Russia

**Keywords:** G-quadruplexes, i-motifs, (immuno)fluorescence microscopy, atomic force microscopy, transmission electron microscopy

## Abstract

The non-canonical structures formed by G- or C-rich DNA regions, such as quadruplexes and i-motifs, as well as their associates, have recently been attracting increasing attention both because of the arguments in favor of their existence in vivo and their potential application in nanobiotechnology. When studying the structure and properties of non-canonical forms of DNA, as well as when controlling the artificially created architectures based on them, visualization plays an important role. This review analyzes the methods used to visualize quadruplexes, i-motifs, and their associates with high spatial resolution: fluorescence microscopy, transmission electron microscopy (TEM), and atomic force microscopy (AFM). The key approaches to preparing specimens for the visualization of this type of structures are presented. Examples of visualization of non-canonical DNA structures having various morphologies, such as G-wires, G-loops, as well as individual quadruplexes, i-motifs and their associates, are considered. The potential for using AFM for visualizing non-canonical DNA structures is demonstrated.

## INTRODUCTION

Hoogsteen binding of nucleotide bases in DNA gives rise to a number of non-canonical structures, including G-quadruplexes and i-motifs [1, 2]. In recent years, evidence has emerged that G-quadruplexes and i-motifs exist in the living cells of various organisms, including humans [3, 4]. These non-canonical DNA structures may be responsible for regulating molecular processes within the cell, including DNA replication, transcription, and genome maintenance [5, 6]. A large number of G-rich sequences in the promoter and telomeric regions of oncogenes (which, therefore, are also C-rich according to the DNA complementarity principle) makes G-quadruplexes (and i-motifs) a potential target for the delivery of antitumor agents into the cell [7 , 8, 9, 10]. In addition, DNA architectures based on G-quadruplexes and i-motifs are in demand in bionanotechnology: for creating molecular machines, developing biosensors and molecular electronic devices, performing molecular diagnostics, etc. [11 , 12, 13, 14, 15, 16].

The conventional methods for detecting and analyzing non-canonical DNA structures include circular dichroism, nuclear magnetic resonance spectroscopy, and UV absorption spectrophotometry during melting [17, 18]. These methods provide characteristics averaged over a large ensemble of molecules (e.g., light absorption or molar ellipticity at certain wavelengths), which allow one to assess the structure of G-quadruplexes and i-motifs. A no less important aspect in the study of the structure and properties of non-canonical DNA structures is their visualization. In addition, direct visualization is required in order to be able to control the generated DNA architectures. However, the nanoscale of the guanine quadruplex quartet or the cytosine–cytosine pair in the i-motif significantly limits the number of methods that can cope with this task.

One of the solutions to this problem is to fluorescently label antibodies against non-canonical DNA structures (immunofluorescence microscopy) or non-canonical DNA structures per se (fluorescence microscopy). These labels make it possible to reveal non-canonical DNA structures in the test sample (e.g., inside the cell) and analyze their distribution. Using this method, non-canonical structures are visualized according to a dot label, which precludes any evaluation of the morphology of the DNA structure per se.

Sufficient spatial resolution for visualizing non-canonical DNA structures is provided by electron and scanning probe microscopy. Meanwhile, scanning electron microscopy, where the image is produced by backscattered electrons, is almost never used for visualizing DNA structures due to a number of inherent limitations, such as the need to study conductive samples and lower resolution compared to transmission electron microscopy. On the contrary, transmission electron microscopy (TEM), which is based on the transmission of an electron beam through an ultrathin (~ 0.1 μm thick) sample, is widely used for studying DNA. In TEM, an image produced by the electrons that have passed through a sample is amplified by electromagnetic lenses and focused on a CCD array.

Scanning probe microscopy (SPM) is a class of methods where image production is based on local interaction between a probe and the sample surface in a large number of points. The most common type of SPM is atomic force microscopy (AFM), which is based on the exchange interaction between probe atoms and a sample [19].

SPM significantly differs from TEM not only in terms of its principle of operation, but also in terms of the sample preparation procedure. Thus, a typical procedure for preparing DNA for TEM examination includes fixing the sample using glutaraldehyde or formaldehyde, as well as creating a contrast by sputtering heavy metal ions onto the sample or treating it with a contrast agent. In addition, electron microscopy studies are usually carried out under vacuum (the low-vacuum models of transmission electron microscopes, which allow one to examine samples in an aqueous vapor or solutions, are characterized by a significantly lower spatial resolution and a complicated procedure of sample preparation and selection of working parameters) [20, 21]. The aforelisted conditions of DNA sample preparation and investigation are far from physiological; therefore, DNA structures visualized using electron microscopy can significantly differ from native ones. In addition, contrasting reduces the resolution of the resulting TEM images. Scanning probe microscopy methods are a more flexible tool in the context of sample preparation conditions and scanning environment as they allow DNA to be deposited from aqueous solutions without additional components alien to the native environment and to conduct the study in air and liquid media [22]. An additional distinguishing feature of AFM is that it allows real-time visualization of dynamic processes [23]. Thanks to these factors, AFM is a method widely used for visualizing various DNA structures and their associates at the level of individual molecules.

This review systematizes the key methods and approaches used to visualize G-quadruplexes, i-motifs and their associates, as well as to analyze the main scientific achievements related to the visualization of these non-canonical DNA structures. The methodological aspects of DNA sample preparation for AFM are also discussed.

## FLUORESCENCE MICROSCOPY OF NON-CANONICAL DNA STRUCTURES

This line of research has recently been intensively developing thanks to the design of small fluorescent molecules (probes) that specifically bind to G-quadruplexes and i-motifs and allow one to localize the latter through fluorescence. For a G-quadruplex, such specific binding can be performed thanks to the π-stacking interaction between a fluorescent dye and the outer tetrad of the G-quadruplex, the interaction between a probe and the loops or grooves of the G-quadruplex, as well as the intercalation of the dye between two quadruplexes [24]. Such ligands are often used in tandem with DNA-duplex-specific fluorophores (e.g., Hoechst dye or propidium iodide), making it possible to compare the localization of canonical and non-canonical DNA structures in one image. One of the quadruplex ligands is 3,6-bis(1-methyl-4-vinylpyridinium)carbazole diiodide (BMVC). For example, it has been used to establish that quadruplexes are formed on the proximal (telomeric) regions of chromosomes [25, 26]. Another fluorescent dye, thiazole orange, is also used to visualize G-quadruplexes [27] and i-motifs [28] thanks to the highly specific binding to them, accompanied by the strong increase in fluorescence. The disadvantage of this dye in the context of the visualization of G-quadruplexes and i-motifs is its low selectivity, as it is able to bind to other nucleic acid structures as well, including double-stranded DNA, three-stranded DNA, and RNA [27, 29, 30]. Another fluorescent dye, thioflavin T (ThT), widely used for specific staining of amyloids, also binds to various DNA structures, while the fluorescence amplification upon binding to G-quadruplexes is especially high (~ 2,100-fold in the visible region) [31, 32]. In recent years, a number of new compounds have been developed and studied in order to be used as fluorescent probes for G-quadruplexes and i-motifs [33], and benzothiazole derivatives in particular [34, 35].

**Fig. 1:**
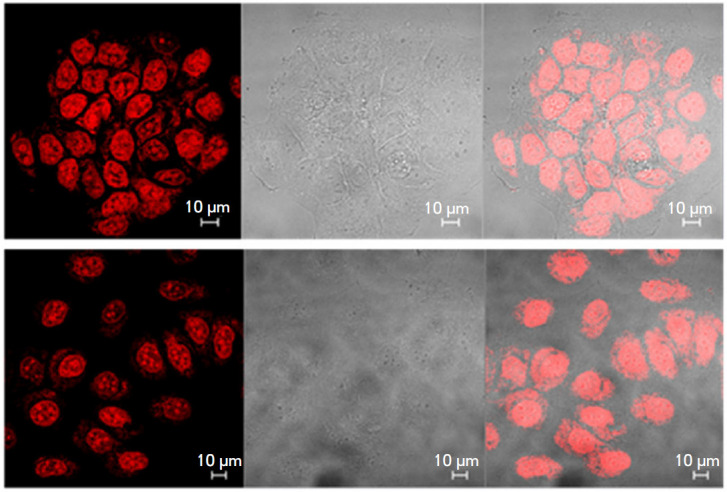
A confocal laser scanning microscopy image of fixed MCF-7 cancer cells labeled (before fixation) for 6 h using cex-NDI, demonstrating nuclear localization of G-quadruplexes (left – fluorescence signal; center – bright field observation; right – superposition of two signals). Reproduced from [37] under the CC 4.0 license (http://creativecommons. org/licenses/by/4.0/)

Because of the supposed participation of G-quadruplexes in gene expression and disease pathogenesis, intracellular visualization of these structures is of particular interest. For a long time, such studies were mainly conducted in fixed cells. Thus, Yan et al. [36] designed a new quadruplex fluorophore, S1, which exhibited high selectivity for binding to G-quadruplexes in in vitro experiments, as well as strong fluorescence in the nucleolus of fixed HeLa cells and weak fluorescence in the remaining portion of the nucleus. The DNA dye 4’,6-diamidino- 2-phenylindole (DAPI), added simultaneously, stained the nucleus more uniformly. This suggested that G-quadruplexes have a nucleolar localization. The localization of G-quadruplexes in the nuclei of MCF- 7 cancer cells was pinpointed using a core-extended naphthalene diimide fluorescent probe (cex-NDI) (Fig. 1) [37].

Probes that allow to visualize quadruplexes in living cells are of the greatest value. Not only should such probes be highly specific to G-quadruplexes, but they also need to have a low ability to bind to proteins and other biological molecules, be capable of passing through the plasma and nuclear membranes, and possess low cytotoxicity [38].

**Fig. 2:**
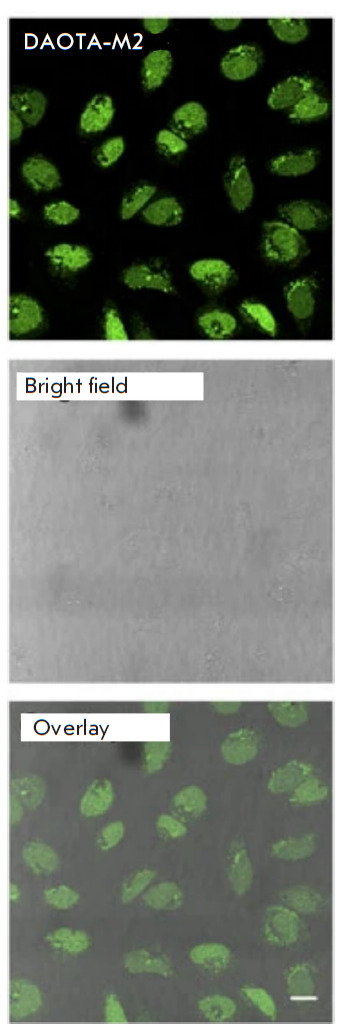
A confocal microscopy image of living U2OS cells incubated with the DOTA-M2 dye, demonstrating nuclear localization of G-quadruplexes (top – fluorescence signal; center – bright field observation; bottom – superposition of two signals). Reproduced from [40] under the CC 4.0 license (http://creativecommons.org/ licenses/by/4.0/)

One of the quadruplex probes used in the fluorescence microscopy of living cells is the BMVC isomer, o-BMVC. Experiments using model objects showed different fluorescence decay times for o-BMVC upon their interaction with G-quadruplexes. Using this fluorophore, G-quadruplexes were localized both in the cytoplasm and in the nucleus of living cells of the CL1-0 line (human lung cancer cells) [39]. Fluorescence lifetime imaging microscopy allowed researchers to both differentiate between duplexes and G-quadruplexes and identify G-quadruplexes of different types, which differ in terms of fluorescence decay time of the ligand bound to them. A similar method – but with a different fluorescent probe (DAOTA-M2) – was used to specify the nuclear localization and stability of G-quadruplexes in living U2OS osteosarcoma cells (Fig. 2) [40, 41]. Nucleolar localization of G-quadruplexes in live MCF-7 cells has recently been confirmed using ThT as a fluorescent probe [42].

Novel fluorophores have recently been developed: they are characterized by a high selectivity to G-quadruplexes, while being highly stable, and can be used in living cells. Examples include such ligands as N-TASQ [43], 2,6-bis((E)-2-(1H-indole-3-yl)vinyl)- 1-methylpyridine-1 iodide [44], carbazole derivatives (4a – 4c) [45], etc. [46, 47]. It has been demonstrated using these probes that G-quadruplexes have nucleolar localization in living cells. N-TASQ has also been used to visualize RNA-based G-quadruplexes in the cytoplasm [48]. Finally, the existence of mitochondrial G-quadruplexes in living cells was shown using fluorescence microscopy [35, 49].

**Fig. 3:**
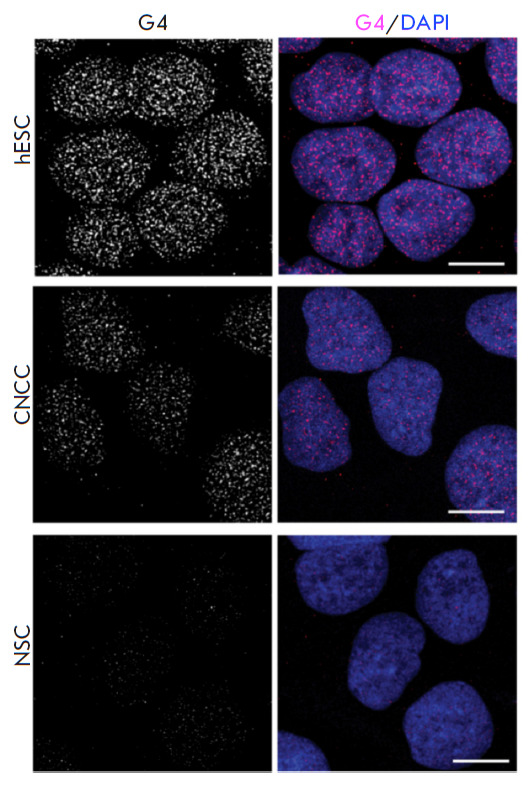
An immunofluorescence microscopy image of fixed pluripotent embryonic stem cells (hESCs), cranial neural crest cells (CNCCs), and neural stem cells (NSCs) labeled with G-quadruplex-specific antibodies BG4 after treatment with RNase (secondary antibodies labeled with fluorescent dye AlexaFluor 488). On the left-hand side, only the quadruplexes are visualized; while on the right-hand side, the nuclei are stained blue due to the contrast with the DNA dye DAPI. The scale bar is 10 μm. Reproduced from [54] under the CC 4.0 license (http://creativecommons.org/licenses/by/4.0/)

An alternative approach to the visualization of G-quadruplexes and i-motifs by fluorescence microscopy is to use specific antibodies enhanced with secondary antibodies tagged with fluorescent labels (immunofluorescence microscopy). For this purpose, various antibodies specific to DNA and RNA G-quadruplexes (e.g., 1H6 and BG4) were synthesized; they allowed one to visualize G-quadruplexes in various cells and tissues [50, 51, 52, 53]. These results are important arguments in favor of the existence of G-quadruplexes in mammalian cells, including the nucleus, cytoplasm, and mitochondria. Thus, immunofluorescence microscopy studies based on BG4 quadruplex antibodies visualized the distribution of G-quadruplexes in the nucleus of hESC pluripotent embryonic stem cells and revealed that the number of G-quadruplexes is significantly reduced during cell differentiation (Fig. 3) [54].

Recently, i-motifs were visualized in vivo in the nuclei and chromosomes in the Bombyx mori testis using immunofluorescence staining with an antibody specifically recognizing the endogenous transcription factor BmILF, which is highly specific to the structure of i-motifs [55].

Meanwhile, the application of immunofluorescence microscopy is hampered by such factors as the relatively high cost of antibodies, as well as their low stability and potential immunogenicity [38]. Therefore, the development and application of G-quadruplex-specific fluorophores remain relevant in our efforts to visualize these structures in living cells.

## TEM IMAGING OF NON-CANONICAL DNA STRUCTURES

Although TEM is commonly used to study DNA and DNA-containing structures in general, this method is quite rarely employed for visualizing non-canonical DNA structures. The typical objects of such visualization are DNA molecules with a non-canonically folded fragment. In particular, TEM has helped discern various loops on double-stranded DNA molecules associated with the formation of G-quadruplexes on one of the two DNA strands.

One of the types of such loops arising after the intracellular transcription of G-rich sites are known as G-loops [56]. G-loops are formed on the plasmid genome in vitro or in Escherichia coli, and they consist of a G-quadruplex on the non–coding DNA chain and a stable RNA/DNA hybrid on the coding DNA chain. In addition, the formation of a specific complex between the G-quadruplex of the G-loop and the mismatch repair factor, the MutSa heterodimer, as well as the formation of a MutSa-mediated synapsis between two DNA strands was observed. The observation of such synapses suggested a mechanism of MutSa operation during class-switch recombination. The so-called R-loops, RNA/DNA hybrids that are formed during the transcription of repetitive motifs (CTG)n, (CAG)n, (CGG)n, (CCG)n and (GAA)n and are associated with some human diseases, also have a structure similar to that of G-loops. R-loops were visualized using TEM [57], but there was no evidence of the formation of G-quadruplexes on a non-coding chain of R-loops. The formation of loops on the G-rich regions of the insulin gene after denaturation and renaturation of the DNA molecule, visualized using TEM [58], was attributed to the formation of quadruplexes on one of the DNA chains.

TEM has also been used to visualize quadruplexes formed in a controlled environment by parallel duplexes bearing G-repeats [59]. The TEM images showed a narrow distribution of the lengths of such structures, consistent with the expected size. In addition, one-dimensional quadruplex-containing nanostructures in the form of nanowires of various lengths are visualized.

**Fig. 4:**
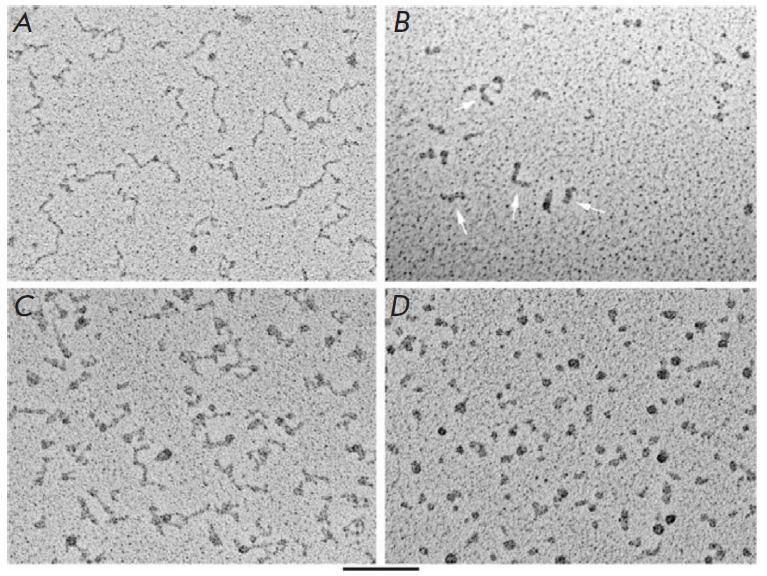
Visualization of C- and G-rich telomeric transcripts. C-rich (*A*) and G-rich (*B*) RNA molecules in 100 mM KCl were deposited for EM on thin carbon substrates, dehydrated, and shaded with tungsten on a rotating substrate. C-rich RNAs look like elongated strands with nodes. G-rich RNAs look like a mixture of balls and sticks (arrows). The rod thickness is significantly greater than that of C-rich or duplex RNAs. C-rich (*C*) and G-rich (*D*) RNA molecules are deposited from 10 mM KCl in the same way as in (*A*) and (*B*). C-rich RNA looks straightened with nodes, while G-rich RNA is mainly ball-shaped. The scale bar is 100 nm. Reproduced from [60] under the CC 4.0 license (http://creativecommons.org/licenses/ by/4.0/)

An analysis of the RNA transcripts of C- and G-rich mammalian telomeric DNA carried out using TEM revealed fundamental differences in their morphologies. C-rich RNA transcripts have a more elongated structure, with a thickness typical of single-stranded RNA, while G-rich transcripts are round particles and short, thick rod-like structures that prevail at elevated salt concentrations (Fig. 4). The observed morphology allowed one to propose a model suggesting that G-rich telomeric RNA is assembled into particle chains, each consisting of four UUAGGG repeats stabilized by parallel G-quartets and connected by UUA linkers [60].

**Fig. 5:**
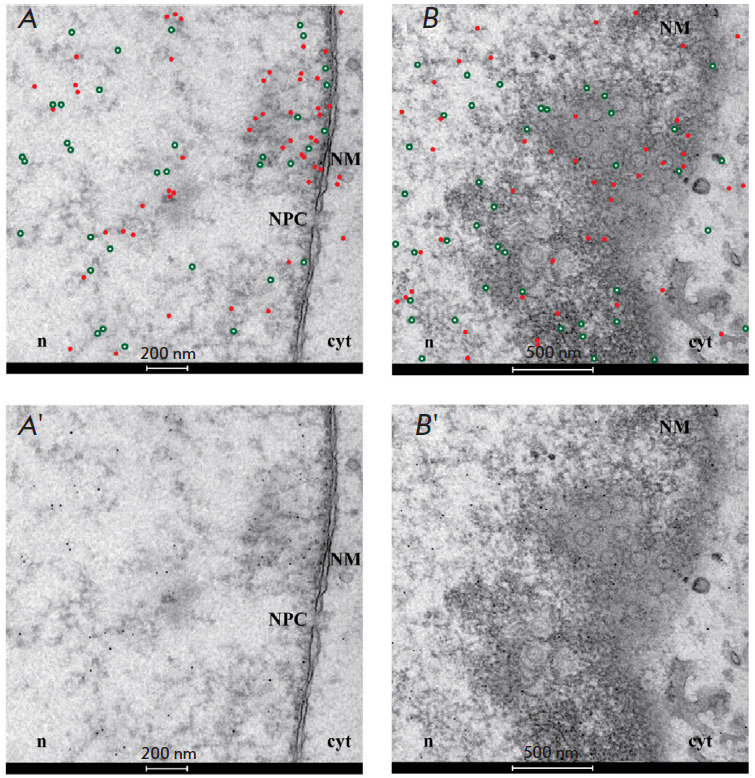
An immunoelectron microscopy image of cells infected with herpes simplex virus type 1 (HSV-1), fixed for 15 h post-infection, and incubated with the anti-G-quadruplex antibody (1H6) and anti-ICP8 serum. Primary antibodies to 1H6 and to ICP8 were detected using gold particles with a diameter of 5 and 10 nm, respectively. To improve image clarity, the golden particles are highlighted with red dots (showing G-quadruplexes) and green circles (showing ICP8) on *Fig. A* and *Fig. B*. The original images are shown in panels *A’ *and *B’*. (*A*-*A*’) G-quadruplexes and ICP8 concentrate in the vicinity of the nuclear membrane (NM), where the nuclear pore complex (NPC) is located. The exit from the nucleus through the nuclear pore complex is one of the pathways used by HSV-1 capsids to leave the nucleus (n) and get into the cytoplasm (cyt). (*B*-*B*’) G-quadruplexes and the ICP8 cluster near the nuclear membrane, where the newly formed virions bud off. Reproduced from [61] under the CC 4.0 license (http://creativecommons. org/licenses/by/4.0/)

With the advent of anti-G-quadruplex antibodies, quadruplexes were also studied using immunoelectron microscopy. In this method, the quadruplexes are detected using TEM imaging of tags (gold nanoparticles) conjugated to secondary anti-G-quadruplex antibodies. Immunoelectron microscopy does not attain the molecular resolution of the quadruplexes per se, but it allows one to observe the distribution of G-quadruplexes inside the cell, which is important for understanding the role played by quadruplexes in intracellular processes. Thus, the formation of G-quadruplexes in cells infected with the herpes simplex virus type 1 was visualized using this method. The formation of G-quadruplexes depended on the stage of the infection cycle: viral G-quadruplexes whose number reached the maximum during virus replication in the cell nucleus moved to the nuclear membrane at the time of virus exit from the nucleus (Fig. 5) [61]. G-quadruplexes in mammalian heterochromatin were detected in a similar way [62]. Therefore, not only do the findings accumulated using immunoelectron microscopy indicate the existence of G-quadruplexes in cells, but they also suggest that G-quadruplexes play a crucial role in biological processes.

## GENERAL APPROACHES TO STUDYING NUCLEIC ACIDS BY AFM

The key component of an atomic force microscope [19] is an elastic plate called a cantilever, with a tip (probe) on it. The interaction between the probe and the surface causes cantilever bending, which is detected by a high-precision optical system consisting of a laser, a photodiode, and the mirror surface of the cantilever. The contact mode of scanning, when cantilever bending serves as a feedback signal and is maintained constant, is rarely used for investigating DNA because of the significant interaction forces between the cantilever and the sample, which cause biomolecule deformation and deteriorate the spatial resolution of the image. AFM studies of biomolecules are typically carried out in the intermittent contact mode [63], when the cantilever oscillates near the resonant frequency, and the interaction between the probe and the sample is determined by the changes in the oscillation amplitude caused by this interaction, which is maintained constant by the feedback. In the intermittent contact mode, the normal forces between the cantilever and the sample are significantly lower than those in the contact mode of scanning and the lateral forces associated with adhesion do not substantially affect the production of the AFM image, since the cantilever periodically “unsticks” from the sample surface, when it moves along the surface during scanning. Over the past decade, the modes based on the periodic approach and withdrawal of the cantilever to/from the surface (the PeakForce mode, “jumping” mode, etc.) have become widely used; these modes can significantly reduce the interaction force between the cantilever and the sample surface [64].

The essential condition for studying DNA using AFM is being able to immobilize a molecule on a substrate. Immobilization of a biomolecule depends on a number of factors such as the composition, pH, concentration of the components of the solution from which the sample is deposited, temperature, the application method, adsorption time, substrate properties, etc. Therefore, sample preparation plays a crucial role in AFM. Smoothness (low roughness) is one of the requirements imposed on the substrate surface for biopolymer deposition. The two most common AFM substrates having areas with atomic smoothness, mica and highly oriented pyrolytic graphite (HOPG), are of a crystalline nature. However, when DNA is deposited onto these surfaces from aqueous solutions, individual molecules in the straightened state are not adsorbed: so, they cannot be studied by AFM.

The reason hindering DNA adsorption on freshly cleaved mica is the similar negative charge of the phosphate groups in the biopolymer and the mica surface, leading to electrostatic repulsion of DNA from the surface. Several strategies have been developed and successfully applied for many years to overcome this phenomenon. The most common one is to use divalent cations such as Mg^2+^, Ca^2+^, Zn^2+^, etc., which act as electrostatic “bridges” between the mica atomic lattice and DNA phosphate groups [65, 66]. In the real world, freshly cleaved mica is pre-modified in an appropriate saline solution before applying DNA or a small amount of this solution (1–10 mM) is applied to mica simultaneously with DNA. Another method, preliminary modification of the mica surface with aminosilanes (e.g., 3-aminopropyltriethoxysilane), is also used to deposit DNA onto mica [67]. In this case, DNA adsorption on the substrate is caused by its attraction to the positively charged amino groups of the modifier. The two strategies described above (the use of divalent cations and aminosilanes) differ in terms of the strength of DNA adsorption: adsorption of molecules mediated by divalent metal cations is relatively weak, enabling thermal motion of DNA near the surface [68 , 69, 70, 71]. On the contrary, mica modified with aminosilanes typically serves as a “kinetic trapping” for DNA; i.e., adsorbed DNA molecules remain immobile on the surface, and their conformation represents the conformation in the solution [72].

A large body of evidence has been accumulated, indicative of the formation of potassium carbonate on the mica surface during its cleavage under laboratory conditions [73]. When immersed into an aqueous solution, the resulting salt can ensure high ionic strength near the mica surface (i.e., just within the area where the main interactions between the biopolymer and the surface occur during its adsorption). This effect, in particular, was observed according to the intense dissociation of the DNA–protein complexes deposited onto mica from a solution with low ionic strength [74]. This characteristic of mica significantly complicates the interpretation of the results obtained for this substrate and, in particular, makes it impossible to perform studies on its surface at low ionic strengths.

Unlike mica, HOPG is electrically neutral and does not form any salts on its surface. However, due to the weak interaction between DNA and graphite, adsorption of DNA molecules in the straightened state onto a freshly cleaved graphite surface is also difficult: DNA is usually adsorbed on this substrate only as aggregates or network structures [75, 76]. A number of approaches based on graphite modification have been developed to overcome this difficulty. The use of modified graphite makes it possible to study DNA at low or zero ionic strengths, which is important for studying the patterns of formation of non-canonical DNA structures.

One of such approaches is to preliminarily modify graphite in a glow discharge in the presence of pentylamine vapors: the surface modified in this way, saturated with amino groups, enables the adsorption of individual straightened DNA molecules onto it, and the dimensions of the biopolymer measured from AFM images (height and width at half-height) are much closer to the native DNA dimensions compared to the size of DNA adsorbed on mica [77, 78]. Later, a methodically simpler method was proposed for modifying HOPG: from an aqueous solution of an oligoglycine derivative N,N’- (decane-1,10-diyl)bis(tetraglycinamide) ([Gly4–NHCH_2_] C8H16[CH_2_NH–Gly4]) known as a graphite modifier or GM [79]. Modification of the HOPG surface with GM, usually carried out by drop casting, gives rise to a homogeneous, self-ordered layer of these molecules with a thickness of less than 1 nm [80, 81]. As is the case with pentylamine modification, GM amino groups make it possible to adsorb individual DNA molecules onto the surface and further study them by AFM [82].

In addition, a number of other organic nanotemplates self-organizing on the graphite surface have recently been used to deposit DNA onto HOPG. Such nanopatterns are formed on crystal surfaces by many alkane derivatives, including stearic acid, dodecylamine, octadecylamine, stearyl alcohol, etc. [83 , 84, 85, 86]. DNA molecules are typically aligned along nanopattern-forming lamellae upon adsorption onto such surfaces [87, 88]. In this case, the chemical nature of the modifier molecule can significantly affect the conformation and properties of the adsorbed DNA molecule [89, 90].

The described approaches for preparing and using DNA samples can also be applied to non-canonical DNA structures, including G-quadruplexes and i-motifs.

## AFM VISUALIZATION OF NON-CANONICAL DNA STRUCTURES

**Fig. 6:**
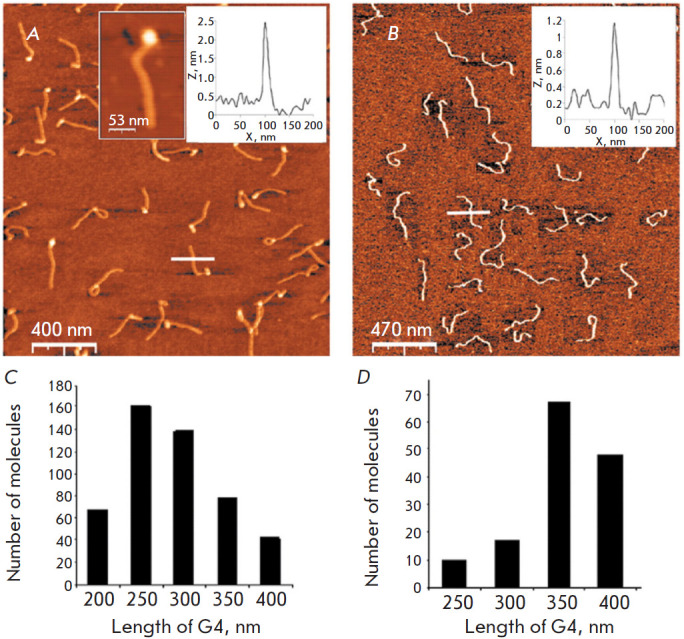
Comparison of tetra- and monomolecular G-quadruplexes. AFM images of tetramolecular (insert: the zoomed-in tetramolecular complex) (*A*) and monomolecular (*B*) G-quadruplexes. Tetramolecular G-quadruplexes were prepared using a complex of four 1,400 bp long 5’-biotin-poly(dG)-poly(dC) molecules associated with avidin. Monomolecular G-quadruplexes were prepared using a 5,500-base-long G-chain. Molecules of both types were deposited on mica under the same conditions. Statistical analysis of the contour lengths of tetramolecular (*C*) and monomolecular (*D*) G-quadruplexes. Reproduced from [95] under the CC 4.0 license (http://creativecommons.org/licenses/by/4.0/)

As noted above, the main factor complicating direct visualization of non-canonical DNA structures without using labels is their small size. The guanine tetrad or double Hoogsteen cytosine dimer has a fixed size, and the number of such G-tetrads or double cytosine dimers in the stack can vary quite widely. The longest non-canonical DNA structure is the G-nanowire: it is formed from G-rich (e.g., poly(G)) nucleotide sequences due to spontaneous formation of a long DNA tetraplex and reaches micron lengths. The morphological characteristics of G-nanowires obtained from AFM images, such as diameter, length, and contour shape, make it possible to determine their structure (e.g., the number of DNA molecules involved in the formation of G-wire), persistent length, and can also be used as feedback when developing procedures for synthesizing these structures for biotechnological applications. A G-nanowire can be formed from a large number of oligonucleotides “interlocking” with each other in a tetraplex [91 , 9,2, 93, 94] from four parallel guanine sequences (a tetramolecular nanowire) [95], as well as from one long molecule of single-stranded DNA folded four times (a monomolecular nanowire) [96, 97]. In the first case, G-nanowires are characterized by a broad length distribution, whereas, in the other two cases, the distribution is narrow. The diameter of the G-nanowire measured according to its height in AFM images is usually ~ 2 nm [91, 95, 96], which, taking into account the effect of height underestimation caused by the interaction of the cantilever with a soft sample [98], is consistent with the size of 2.8 nm obtained from a X-ray diffraction analysis of the G-tetrad [99]. The examples of AFM images of G-nanowires are shown in Fig. 6.

Other nanowires based on non-canonical structures have also been described. Thus, hybrid nanowires consisting of fragments of G-nanowires and i-motifs have been synthesized [100]. AFM revealed the polymorphism of such structures, which depends on the oligonucleotides used and the ionic environment, in particular, the supramolecular conformation of the hybrids, as well as the V-shaped, circular and linear configuration of the hybrids. Fibrils 0.45–4 nm high and up to 2 μm long were also found to form from two types of oligonucleotides: SQ1A (CAGTAGATGCTGCTGAGGGGGGGTGTGTCTTCAAGCG) and SQ1B (CTCTACGACGACTGGGGGGGACACGAAGTTCGCTACTG), which is attributed to the formation of numerous synapses based on quadruplexes [101].

Thanks to the possibility of feeding an electric potential to the cantilever of an atomic force microscope and measuring the current, the current–voltage curves of individual G-nanowires can be recorded. In particular, it has been shown that the G-nanowire is capable of conducting current from several tens to several hundreds of picoamps [11].

In nature, G- or C-rich nucleotide sequences prone to forming non-canonical structures are usually embedded in longer DNA molecules (e.g., the telomeric regions of chromosomes or near the promoter). In double-stranded DNA, G- and C-rich motifs always reside opposite to each other due to complementarity. Thus, AFM allowed one to visualize the simultaneous formation of a G-quadruplex and an i-motif on double-stranded DNA containing a G-rich VNTR motif: CGC(GGGGCGGGG)n. These structures had a branched shape and were observed only in an acidic medium and in the presence of K+ ions. The formation of a G-quadruplex and an i-motif in the VNTR sequence can occur during transcription or replication, when double-stranded DNA becomes single-stranded and, thus, affects the expression of the respective gene [102].

To study the formation of non-canonical structures and visualize them, G- and C-rich motifs are often “embedded” in a DNA molecule or a DNA-based nanostructure with a size much larger than that of the non-canonical structure per se. Therefore, it becomes possible to identify individual G-quadruplexes or i-motifs by changing the morphology of the larger DNA structures connected to them.

Thus, a single-stranded DNA region containing two C-tracts was inserted into a double-stranded circular DNA region from opposite sides of the “ring” [103]. The formation of various intra- and intermolecular i-motifs was shown, in particular, using AFM visualization of the architecture of the DNA rings. The mutual arrangement of these rings also made it possible to determine the role of the length of the C-repeats of a single-stranded DNA region in the formation of intra- or intermolecular i-motifs: the presence of two tracts of six or less cytosine bases gave rise to an intermolecular i-motif, while a larger number of repeats of cytosine tracts yielded an intramolecular i-motif.

**Fig. 7:**
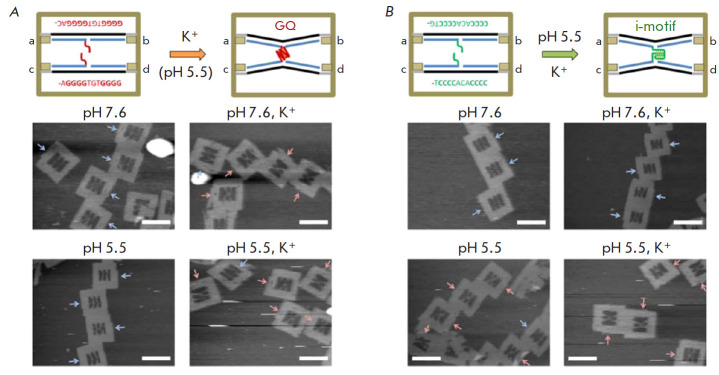
Observation of the formation of a separate G-quadruplex and i-motif. (*A*) – AFM images of DNA frames containing the G-tracts required for the formation of an interstrand G-quadruplex in the presence of K+. (*B*) – AFM images of DNA frames containing the C-tracts required for the formation of an interstrand i-motif in an acidic medium. The blue and red arrows show disconnected and connected (X-shaped) threads, respectively. The scale bars are 100 nm. Adapted with permission from [105]. Copyright (2015) American Chemical Society

For performing AFM visualization of the formation of individual non-canonical DNA structures, it was proposed to embed the G- and C-rich sequences of oligonucleotides into a rectangular DNA origami frame. Using high-speed AFM, the formation and dissociation of the G-quadruplex inside such a frame are visualized in real time. Meanwhile, the formation and dissociation of G-quadruplexes was identified according to the changes in the contours of two DNA molecules carrying a G-quadruplex sequence from the parallel to the X-shaped one (during the formation of a G-quadruplex), and vice versa (during its dissociation) [104]. Topologically controlled G-quadruplexes and i-motifs were formed on the basis of the DNA-nanoframe by moving the DNA chain, adding or removing K+ ions, and using an acidic medium. Dissociation of double-stranded DNA with the formation of a G-quadruplex and an i-motif was visualized by high-speed AFM (Fig. 7) [105].

Investigation of individual short G- or C-rich oligonucleotides capable of forming non-canonical DNA structures, as well as their interaction with each other, is of no less fundamental and practical interest. AFM visualization of such sequences allows one to supplement the data obtained using the conventional methods for studying quadruplexes (such as circular dichroism, thermal melting, NMR, etc.) with the morphological parameters of individual structures and their statistical distribution. Nevertheless, AFM studies of non-canonical DNA structures consisting of short oligonucleotides are quite rare.

An AFM study of a synthetic oligonucleotide adsorbed onto a mica surface containing a G-rich CpG motif revealed that there are heterogeneous structures 1–6 nm high which most likely are the G-quadruplexes and their aggregates [106]. AFM visualization of oligonucleotides consisting of 16 telomeric TTAGGG repeats allowed one to infer that most of these oligonucleotides form only two quadruplexes out of the four possible ones, which resemble beads on a string in the AFM images [107]. G-quadruplexes of the oligonucleotides d(G)_10_, d(TG_9_), and d(TG_8_T) were formed only in a Na^+^-containing solution with a long incubation time or in a K^+^-containing solution and looked like spherical aggregates 1.5–3 nm high or nanowires (for d(G)_10_) [94].

AFM imaging can also be used to study the interaction between individual non-canonical DNA structures and various ligands. Investigating this interaction would be of great relevance because of the suspected role of G-quadruplexes in many intracellu lar processes, as well as their potential use as targets for antitumor drugs.

For example, when studying the interaction between G-quadruplexes and polyamines, aggregation of G-quadruplexes was visualized: the height of the observed structures increased from 3 to 4–11 nm, depending on the type of polyamine [108]. Another series of AFM experiments showed that the triazole-linked acridine ligand GL15 binding to G-quadruplexes stabilizes and accelerates the formation of quadruplexes in Na^+^- and K^+^-containing solutions [109]. It was also shown that prolinamide derivatives can selectively bind and stabilize G-quadruplexes. An AFM study showed that the tris-prolinamide derivative Pro-4 can drive the formation of structures from G-quadruplexes based on c-MYC [110].

The small number of AFM studies of individual G-quadruplexes formed from oligonucleotides and their associates infers that the size of these structures being close to the resolution limit of AFM in soft objects is the main roadblock in such studies. The spatial resolution of an atomic force microscope depends on a number of factors, including the cantilever tip radius. Therefore, one of the ways to increase the resolution of AFM images is to use super sharp cantilevers. Cantilevers with a radius of curvature of down to 1 nm are commercially available today [79, 111]. The nature of the substrate can also limit the resolution of an atomic force microscope: for example, the formation of a salt film on the mica surface can reduce the height of the adsorbed DNA structures in AFM images, thus worsening the contrast and reducing the spatial resolution.

**Fig. 8:**
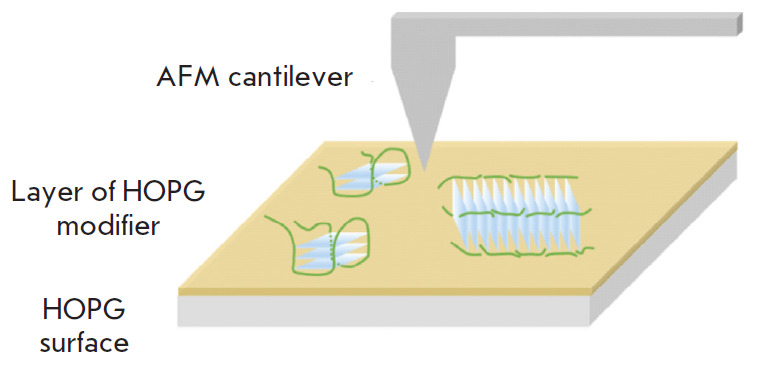
Schematic representation of the sample surface for AFM studies of the quadruplexes and quadruplex-containing structures on the surface of the GM modified HOPG

**Fig. 9:**
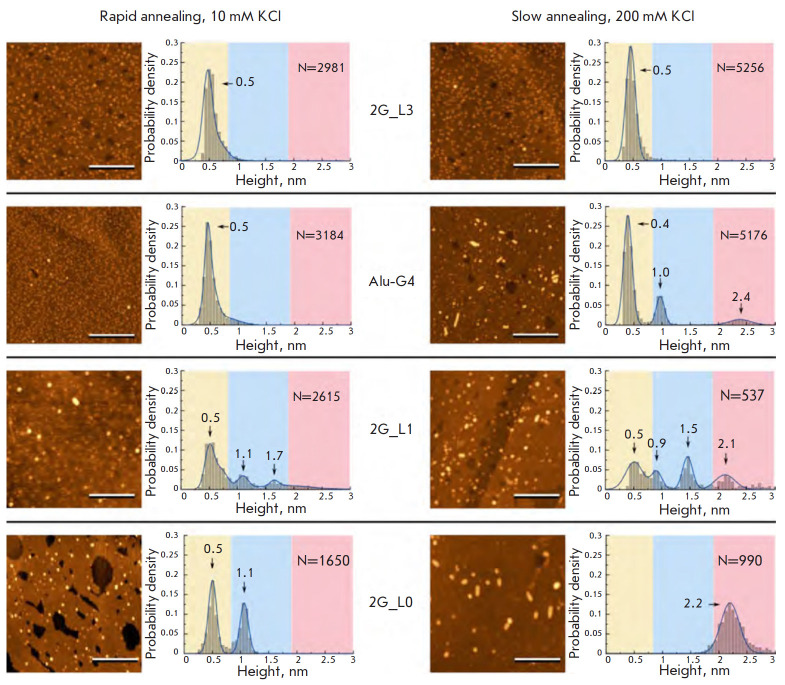
AFM images and corresponding histograms of the height distribution of two tetrad G-quadruplexes formed after rapid annealing in the presence of 10 mM KCl (left-hand side) and slow annealing in the presence of 200 mM KCl (right-hand side). The scale bar is 100 nm. Reproduced from [112] under the CC 4.0 license (http://creativecommons.org/ licenses/by/4.0/)

The use of ultrasharp cantilevers and the GM-modified HOPG’s surface as a substrate often allows one to improve the quality of a structural analysis of biomolecules using AFM [79, 80]. This approach, illustrated in Fig. 8, has been applied in several studies of non-canonical DNA structures. The polymorphism of quadruplexes formed under different conditions from G-rich oligonucleotides with different lengths of the G-tracts and loops between them was analyzed by AFM [112]. An analysis of the morphology and histograms of the heights distribution of the visualized structures allowed one to distinguish from one to four types of the quadruplexes formed by each of the oligonucleotides and identify the patterns of formation of molecular associates (multimers of G-quadruplexes) from intramolecular G-quadruplexes (Fig. 9).

**Fig. 10:**
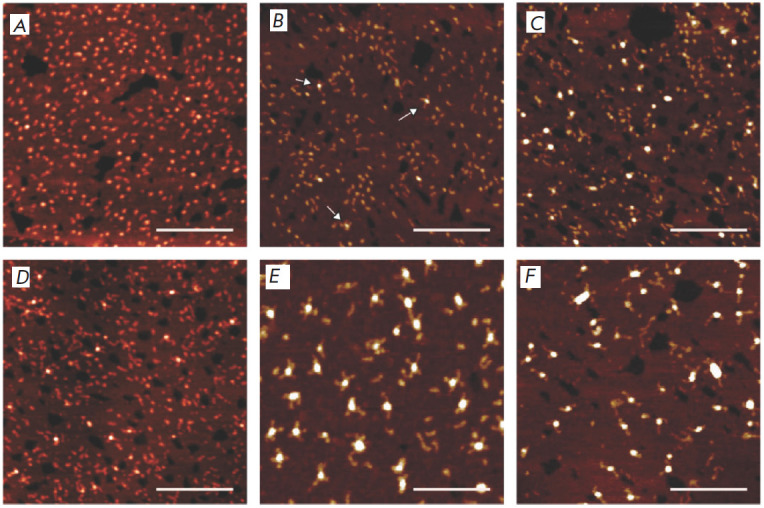
AFM images of the nanostructures based on i-motifs formed by the following oligonucleotide sequences at pH 5.5: C2T25 (*A*); C5T25 (*B*); C7T25 (*C*); C9T25 (*D*); C12T25 (*E*); and C25T25 (*F*). The scale bar is 100 nm. Reproduced from [114] under the CC BY-NC 3.0 license (https:// creativecommons.org/ licenses/by-nc/3.0/)

It has been shown using model oligonucleotides (fragments of the human genome containing a G/C-rich region in the middle) using AFM that synaptic contacts between DNA molecules emerge due to the formation of intermolecular G-quadruplexes or i-motifs [113]. The emergence of intermolecular i-motifs explains the structures formed by single-stranded CnT25 oligonucleotides (n = 2, 5, 7, 9, 12, 25) at pH 5.5 and visualized by AFM (Fig. 10) [114]. Such structures consist of the i-motif “core” and the “arms” emerging from it. These structures may be of interest in bioengineering for synthesizing DNA-based molecular architectures.

## CONCLUSIONS

The main methods currently used to visualize non-canonical DNA structures include fluorescence microscopy, TEM and AFM. Fluorescence microscopy allows one to visualize G-quadruplexes, including those in living cells, while visualization is carried out thanks to fluorescent label binding to the DNA-quadruplex. Therefore, much attention is paid to the development of fluorophores with high selectivity to G-quadruplexes and good optical properties. Among the types of fluorescence microscopy used to visualize quadruplexes, fluorescence lifetime imaging microscopy and immunofluorescence microscopy are worth noting. Anti-G-quadruplexes antibodies enhanced by secondary antibodies with fluorescent labels attached are used in the latter case. Over the past few years, fluorescence microscopy has provided a large amount of data proving that G-quadruplexes exist in living cells, with DNA quadruplexes localized mainly in the nucleolus; and RNA quadruplexes, in the cytoplasm.

Unlike fluorescence microscopy, TEM and AFM can help visualize non-canonical DNA structures without using labels. Both methods are characterized by a comparable lateral resolution, while AFM, unlike TEM, has a high height resolution. A number of structures based on non-canonical structures, such as G-loops, R-loops, and G-nanowires, have been visualized by TEM and AFM. Special approaches employing DNA origami nanoframes, in which oligonucleotides capable of forming noncanonical DNA structures are embedded, have been developed for real-time AFM visualization of the formation and dissociation of individual quadruplexes and i-motifs. These studies have allowed us to understand better the influence of conditions, such as the composition and concentration of ions, pH, the distance between interacting DNA fragments, etc., on the formation of G-quadruplexes or i-motifs. AFM and TEM visualization of individual non-canonical DNA structures and their smaller derivatives is the most challenging problem from the methodological standpoint, since the size of such structures is close to the resolution of these methods. AFM resolution for studying non-canonical DNA structures can be further increased by using special substrates (e.g., modified graphite) and ultrasharp AFM cantilevers. This approach has helped visualize the polymorphism of G-quadruplex structures and also detect the emergence of synaptic contacts between oligonucleotides thanks to the formation of intermolecular non-canonical DNA structures.

## Abbreviations

TEM: transmission electron microscopy
BMVC: 3,6-bis(1-methyl-4-vinylpyridinium)carbazole diiodide
AFM: atomic force microscopy
DAPI: 4’,6-diamidino-2-phenylindole
GM: N,N’-(decane- 1,10-diyl)bis(tetraglycinamide)
SPM: scanning probe microscopy
ThT: thioflavin T
